# The efficacy of Narrative Exposure Therapy for Children (KIDNET) as a treatment for traumatized young refugees versus treatment as usual: update to the study protocol for the multi-center randomized controlled trial YOURTREAT

**DOI:** 10.1186/s13063-022-06288-8

**Published:** 2022-04-27

**Authors:** Jasmin Wittmann, Melissa Groß, Claudia Catani, Telja Schmidt, Sina Neldner, Sarah Wilker, Theodor May, Verena Ertl, Rita Rosner, Areej Zindler, Michael Odenwald, Frank Neuner

**Affiliations:** 1 Clinical Psychology and Psychotherapy, Bielefeld University, Universitätsstraße 25, D 33615 Bielefeld, Germany; 2 Independent Biostatistician, Johann-Strauß-Str. 11a, D 33647 Bielefeld, Germany; 3 Clinical Psychology and Biopsychology, Catholic University Eichstätt-Ingolstadt, D 85071 Eichstätt, Germany; 4 Outpatient Clinic for Refugee Children and Adolescents, Medical Center Hamburg-Eppendorf, Martinistraße 52, D 20246 Hamburg, Germany; 5 Department of Psychology, Konstanz University, Universitätsstraße 10, D 78464 Konstanz, Germany

**Keywords:** Post-traumatic stress disorder, Children, Adolescents, Refugees, Narrative Exposure Therapy for Children (KIDNET), Randomized controlled trial

## Abstract

**Background:**

The trial YOURTREAT aims to compare the pragmatic, short-term psychotherapy Narrative Exposure Therapy for Children (KIDNET) with treatment as usual (TAU) for the treatment of young refugees in Germany. This update outlines changes made to the study protocol in response to the current COVID-19 pandemic with the aim of allowing the continuation of the clinical trial while ensuring the safety of the staff and the participants, maintaining methodological quality, and ensuring compliance with legal regulations.

**Methods:**

The major amendments to the original study protocol include (1) the possibility of using telehealth technology for the conduction of diagnostic and therapy sessions, (2) a reduction of the diagnostic set, and (3) an increased flexibility in the time frame of the study protocol.

**Discussion:**

The adaptations to the study protocol made it feasible to continue with the trial YOURTREAT during the COVID-19 pandemic. Although the diagnostic set had to be shortened, the primary outcomes and the main secondary outcomes remain unimpaired by the amendment. Therefore, we expect the trial to provide evidence regarding effective treatment options for young refugees in Germany, a population that has received little scientific attention so far and has only very limited access to mental health care in the German health care system. In light of the current pandemic, which globally increases the risk of mental problems, the situation for young refugees is likely to aggravate further. Thus, the clinical and social relevance of the present trial YOURTREAT is even more important in these particular times.

**Trial registration:**

German Clinical Trials Register (Deutsches Register Klinischer Studien; DRKS) DRKS00017222. Registered on May 15, 2019

## Background

This update refers to the study protocol of the multi-center clinical trial “The efficacy of Narrative Exposure Therapy for Children (KIDNET) as a Treatment for Traumatized Young Refugees versus Treatment as Usual” (YOURTREAT) originally published in *TRIALS* [1] and should be read in conjunction with the original study protocol.

The current COVID-19 pandemic is an unpredicted and extraordinary situation, posing challenges to clinical trials and requiring adaptations of the study protocols for the yet uncertain duration of the pandemic in order to allow for a safe continuation of trial activities and avoid attrition of participants [2]. The following protocol amendment was thus implemented to the clinical trial YOURTREAT as a reaction to the current challenges with the aim of allowing the continuation of the clinical trial while ensuring the safety of the staff and the participants, maintaining methodological quality and ensuring compliance with legal regulations.

In March 2020, German authorities have issued far reaching measures in response to the COVID-19 pandemic including restrictions such as curfew, contact ban, and a prohibition of clinical research. These measures required a temporary suspension of recruitment activities in all study centers from March to July 2020; however, the clinical activities with participants already enrolled in the study (ongoing diagnostic interviews and therapies) were continued for ethical reasons. Because the federal states are responsible for the anti-pandemic regulations in Germany, the specific local requirements vary between the study centers. During the early stage of the pandemic, regulations have been changing almost every 2 weeks and have remained unsteady to the present day. Therefore, the YOURTREAT trial protocol needed to be adapted to ensure the continuation of the trial in these unstable conditions.

### (1) Use of telehealth technologies

In order to allow for the safe continuation of the trial during the pandemic and to make it feasible to reach out to patients who are unable to attend personally (e.g. due to quarantine or travel restrictions), telehealth technologies have been introduced to the trial. Telehealth technologies must be certified for the use in the German health care system and employed in full concordance with the guidelines for video-based therapy issued by the German federal chamber of psychotherapists [3] and the respective currently valid guidelines of the regional chambers of the federal states. In addition, participants in the YOURTREAT project are required to have a telephone connection in place (e.g. mobile phone) and to provide the clinician with the telephone number for the event of disruptions in the internet connection. Before conducting a telehealth-based appointment, the following conditions must be met: (1) the participant has been informed about the nature and particularities of a telehealth-based appointment and an additional informed consent for the application of telehealth has been obtained. (2) Acute suicidality has been ruled out through the evaluation of the qualified clinician. In case either (1) or (2) does *not* apply, the appointment cannot be conducted via telehealth.

Diagnostic interviews can be conducted using telehealth technologies at all time points of the study (t1: pre therapy, t2: 6-month follow-up, t3: 12-month follow-up). In order to avoid a high variability in treatment provision, which might limit the interpretability of the trial results, the use of telehealth technologies for KIDNET therapy sessions should be the exception. Instead, KIDNET should be provided face-to-face following strict hygienic measurements to reduce the risk of an infection. However, telehealth can be allowed in special cases that need to be justified (e.g. to ensure the completion of a therapy during contact-ban, for cases that belong to a high risk group, for cases in quarantine). In case an interpreter is required for a diagnostic and/or therapeutic process, he or she can join the session using telehealth if the participant agrees and if this is perceived as practicable by both therapist and patient.

### (2) Reduction of the diagnostic set

**Fig. 1:**
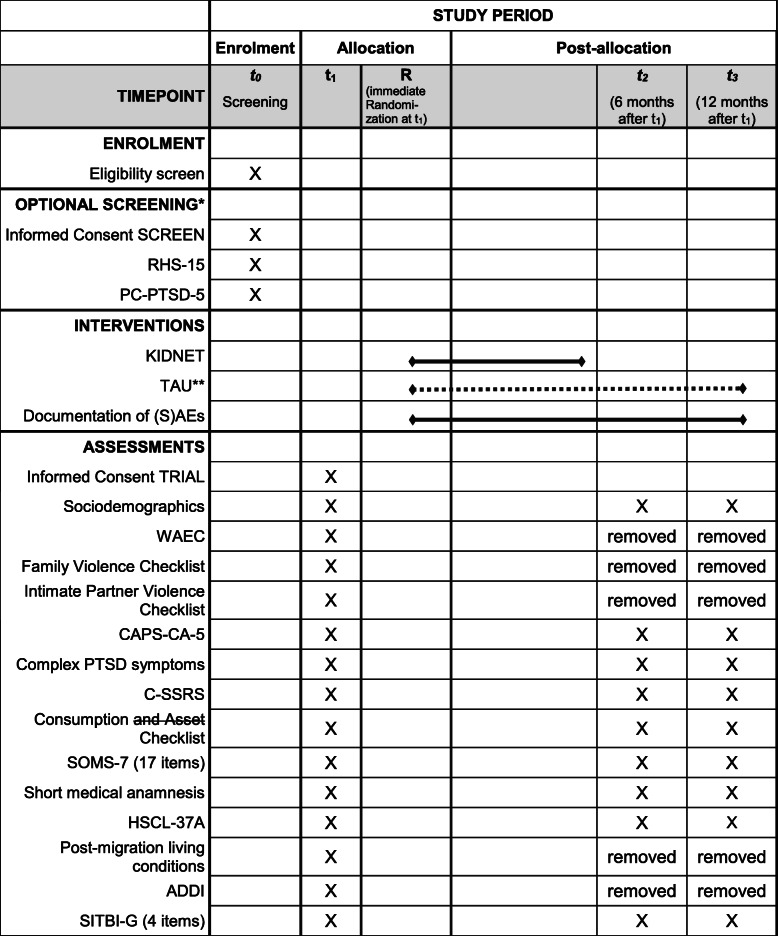
Displayed are the standard protocol items (SPIRIT) for the clinical trial YOURTREAT including enrolment, diagnostic assessments, and interventions—reduction to the most essential elements. -ADDI, Adolescent Discrimination Distress Index; CAPS-CA-5, Clinician-Administered PTSD Scale for DSM-5 – Child/Adolescent Version; C-SSRS, Columbia Suicide Severity Rating Scale; HSCL-37a, Hopkins symptom checklist-37 for refugee adolescents; KIDNET, Narrative Exposure Therapy for Children; PC-PTSD-5, Primary Care Post-traumatic Stress Disorder Screen for DSM-5; RHS-15, Refugee Health Screener-15; (S)AE, (serious) adverse event; SITBI-G, German version of the self-injurious thoughts and behaviors interview; SOMS-7, Screening for Somatoform Symptoms 7; TAU, treatment as usual; WAEC, War and Adversities Exposure Checklist. Single asterisk (*) indicates the following: next to established recruitment procedures of the study centers, participants can be recruited and screened by Intercultural Therapy Assistants as an entryway into the trial. Double asterisk (**) indicates the following: TAU: patients will be instructed to seek treatment within the general health care system. Therefore, in contrast to KIDNET, the duration of TAU cannot be specified a priori

To account for the additional logistical efforts of diagnostic procedures provided through telehealth, some items and instruments have been removed from the diagnostic batteries. For the t1 assessment, single items from the sociodemographic information, mobile phone use, and the asset list have been removed. For t2 and t3 measurements, the same questions have been removed, as well as the event checklists and the questionnaires post-migration living conditions and Adolescent Discrimination Distress Index (ADDI) (see Fig. 1 for an overview). Although the diagnostic set had to be shortened, the primary outcomes and the main secondary outcomes remain unimpaired by the amendment.

### (3) Increased flexibility in the time frame of the trial

To account for the uncertain and unsteady conditions of the trial during the pandemic which are likely to result in delays in the therapeutic or diagnostic processes, we increased the flexibility of the time frame of the trial. The tolerance window of assessments at the post and follow-up timepoints is extended to 4 weeks before until 4 weeks after the scheduled timepoint. The tolerance window for the beginning and ending of the NET has been extended from 1 to 2 weeks. However, the uncertain conditions of the trial may require deviations even from the extended time windows in single cases. If an assessment or therapy appointments should not be possible within the time frame, the procedure should be completed with minimum delay. The reasons for the deviation as well as the details of the delay need to be documented.

## Impact of the COVID-19 pandemic on the trial

At the beginning of the pandemic, in March 2020, the trial was in the initiation phase: Two out of four study centers had already started recruitment, while the remaining two centers prepared for the start of recruitment. A total number of 15 participants had been randomized, and four 6-month follow-up assessments had been completed. Since recruitment activities (e.g., outreach at school, youth centers or refugee accommodations) represented main activities at this stage of the trial, the restrictions due to the pandemic significantly slowed down recruitment into the trial. Therefore, we applied for an extension of the trial activities for 2 years, which was approved by the German Federal Ministry of Education and Research (BMBF).

## Discussion

The aim of the amendment of the clinical trial YOURTREAT was to allow the continuation of the trial after a careful risk assessment. The current pandemic and the corresponding protocol amendments might increase unsystematic variance in the trial. However, since the treatment under investigation, KIDNET, will be conducted face-to-face as originally planned (except for singe cases that need to be justified), the interpretability of the trial results will not be compromised by the protocol changes.

Further, COVID-19-associated impacts on the trial (e.g. canceled appointments due to COVID-19 infections or quarantine of patients) will be carefully documented in order to allow us to investigate the potential impact of the pandemic on the trial.

Given that the mental health burden is globally increased by the pandemic [4] and the fact that refugees have a higher probability to be exposed to COVID-19-associated risk factors such as isolation, crowded housing, economic insecurity, or familial violence [5], the importance of short and pragmatic interventions for this population has been escalated by the pandemic. Therefore, the results of the trial YOURTREAT are urgently needed to inform the regular health care system on effective treatment options for the particularly vulnerable group of young refugees.

## Data Availability

Not applicable
